# Atypical COVID-19 dynamics in a patient with mantle cell lymphoma exposed to rituximab

**DOI:** 10.1186/s13027-021-00376-1

**Published:** 2021-06-02

**Authors:** Gianpaolo Marcacci, Giuseppe Fiorentino, Francesco Volzone, Umberto Falcone, Roberto Parrella, Daniela Donnarumma, Silvia D’Ovidio, Anna Annunziata, Giovanni Micallo, Giuseppe Portella, Annarosaria De Chiara, Rosaria De Filippi, Stefania Crisci, Antonio Pinto

**Affiliations:** 1 Hematology-Oncology and Stem Cell Transplantation Unit, Istituto Nazionale Tumori, Fondazione ‘G. Pascale’, IRCCS, Naples, Italy; 2 Respiratory Physiopathology and Rehabilitation Unit, AORN dei Colli, Naples, Italy; 3 Respiratory Infectious Disease Unit, AORN dei Colli, Naples, Italy; 4 Department of Translational Medical Sciences, Università degli Studi Federico II, Naples, Italy; 5 Pathology Unit, Istituto Nazionale Tumori, Fondazione ‘G. Pascale’, IRCCS, Naples, Italy; 6 Department of Clinical Medicine and Surgery, Università degli Studi Federico II, Naples, Italy

**Keywords:** Mantle cell lymphoma, COVID-19, Rituximab, Anti-CD20 antibodies

## Abstract

Patients with non-hodgkin lymphomas (NHL) represent a population of special interest during the current Coronavirus disease-19 (COVID-19) pandemics. NHLs are associated with disease- and treatment-related immunodeficiencies which may generate unusual COVID-19 dynamics and pose unique management challenges. We report the unusual clinical course of COVID-19 in a patient with mantle cell lymphoma (MCL) exposed to nine doses of Rituximab shortly before infection with severe acute respiratory syndrome corona virus 2 (SARS-CoV-2). He had a prolonged asymptomatic phase, with negative molecular and antibody testing for SARS-CoV-2, followed by a rapidly progressive evolution to severe COVID-19. Despite detection of viral RNA overlapped with first symptoms occurrence, anti-SARS-CoV-2 antibodies displayed an asynchronous pattern, with IgG first appearing 2 days after RNA positivity and IgM never being detected throughout the entire clinical course. While disease-associated immune derangements and/or previous treatments involving anti-CD20 antibodies might have contributed to COVID-19 dynamics in our patient, data suggests that antibody testings, without concurrent molecular assessment for SARS-CoV-2, may turn inadequate for monitoring of MCL patients, and in general NHL patients heavily exposed to anti-CD20 antibodies, during the current pandemics. We suggest that repeated molecular testing of nasopharyngeal swab should be implemented in these subjects despite a negative serology and absence of symptoms of SARS-CoV-2 infection. For the same reasons, a customized strategy needs to be developed for patients exposed to anti-CD20 antibodies, based on different features and mechanism of action of available SARS-CoV-2 vaccines and novel vaccinomics developments.

## Introduction

Shortly after emergence of the Coronavirus disease-19 (COVID-19) epidemics in China, it has been suggested that cancer patients may represent a highly vulnerable group to severe acute respiratory syndrome corona virus 2 (SARS-CoV-2)-related morbidity and mortality [1]. Some investigators, challenged such a view highlighting that age, gender and comorbidities, rather cancer diagnosis itself and/or recent exposure to anticancer treatments, may act as major drivers for increased mortality risk upon SARS-CoV-2 infection [2, 3].

While efforts are ongoing to further elucidate the association between malignancies and COVID-19, specific data on outcomes of patients with non-Hodgkin lymphoma (NHL) are still limited. A study of 128 Chinese patients with hematologic malignancies did not identify any COVID-19 case among subjects with NHL [4]. Differently, NHL cases were described in cohort studies from western countries [5–7] and a very recent report on 536 patients with different types of hemopoietic malignancies, included a significant proportion of NHL cases, supporting that these patients represent a high-risk population with poor COVID-19 outcomes, also when compared to patients with solid cancers [8].

In these studies, however, clinical courses of patients with specific lymphoma subtypes were not always detailed, hampering a thorough assessment of COVID-19 outcomes across the substantial biologic and clinical heterogeneity, including different therapeutic settings, across various NHL entities.

On the other hand, NHLs are associated with disease-related immunodeficiencies, which may render these patients especially susceptible to SARS-CoV-2 infection [9]. In addition, treatments for B-cell NHL typically involve prolonged use of anti-CD20 antibodies, such Rituximab or obinutuzumab, and alkylators, known to induce a severe and prolonged B- and T-cell lymphodepletion, both established risk factors for COVID-19 outcomes [1, 4, 7, 10, 11].

Here, we describe the unusual features of SARS-CoV-2 infection occurred in a patient with mantle cell lymphoma (MCL), a rare NHL lymphoma subtype whose biologic features along with a significant previous exposure to Rituximab might have concurred, at least in part, to the atypical COVID-19 dynamics, evolution and antiviral immune responses.

## Case report

**Fig. 1:**
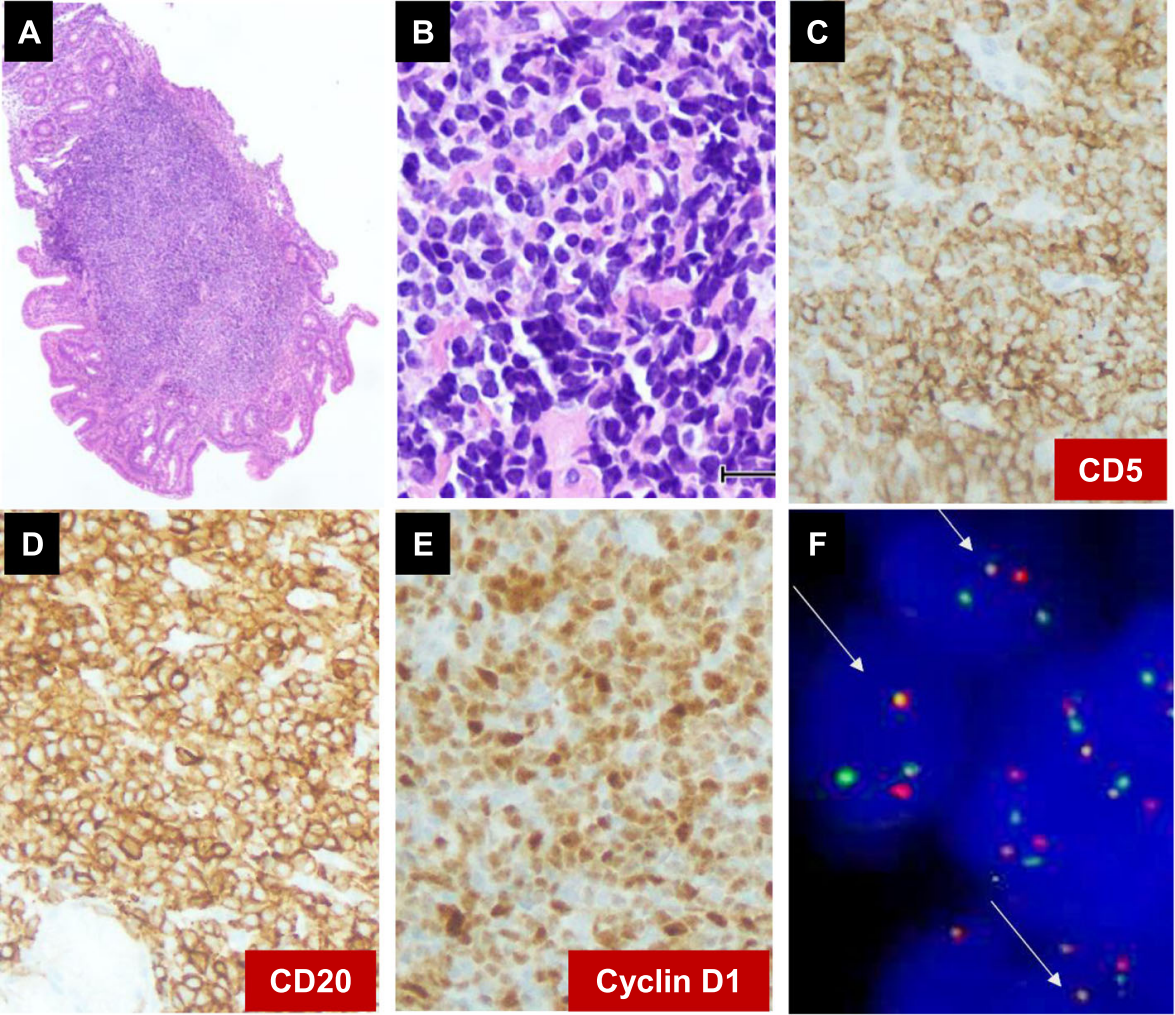
Histopathologic, phenotypic and molecular features of a mantle cell lymphoma case developing COVID-19. **a** and **b** Haematoxylin and eosin stain of duodenal biopsy at restaging (March 11, 2020). **c** CD5, (**d**) CD20 and (**e**) cyclin D1 immunostainings. **f** FISH analysis documenting the presence in tumor cells at restaging of t (11;14) (q13;q32) translocation with IGH-CCND1 fusion. Tumor cells were were CD10 and SOX-11 negative and Kì67 staining was < 10% (not shown)

**Fig. 2:**
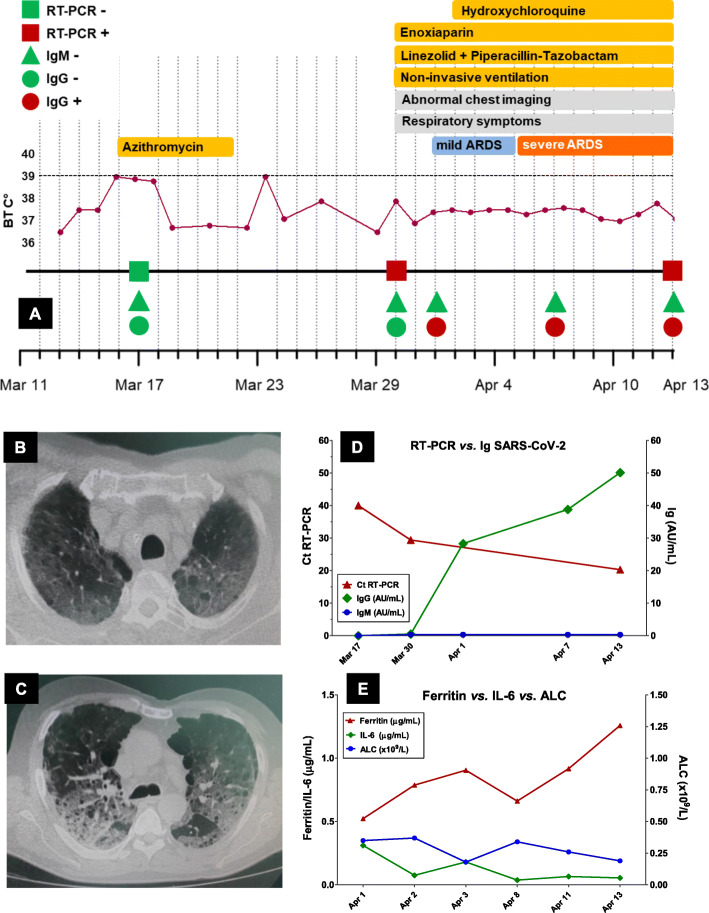
Visual timeline including major clinical findings, treatments, virologic and laboratory data. **a** Clinical timeline and treatments in the context of results for SARS-CoV-2 RT-PCR and virus-specific IgG and IgM antibodies. ARDS severity was scored according to the Berlin definition. **b**, **c** Chest computed tomography scans (April 1, 2029) showing typical features of COVID-19 pneumonia with bilateral consolidative abnormalities, central and peripheral ground glass opacities. **d** RT-PCR results (Ct) vs. detection of anti SARS-CoV-2 IgG and IgM antibodies. RT-PCR data was obtained by using the Allplex™ 2019-nCoV multiplex Assay. IgG and IgM were evaluated by chemoluminiscence immunoassay (Shenzhen YHLO Biotech Co, Ltd) and results expressed in arbitrary units (AU)/mL. Cut off value for positivity as indicated by manufacturer was of 10 AU/mL both for IgM and IgG antibodies (**e**) Changes in serum levels of ferritin, IL-6 and ALC. Abbrevations used in the figures. FISH, fluorescence in situ hybridization; RT-PCR, reverse transcriptase-polymerase chain reaction (RT-PCR); BT, body temperature (°C); ARDS, acute respiratory distress syndrome; AU, arbitrary units; ALC, absolute lymphocyte counts; Ct, cycle threshold

A 71-year-old man was diagnosed stage IVA mantle cell lymphoma (MCL) in September 2019. Disease involved gastro-duodenal tract, paratracheal, intra-abdominal and inguinal lymph nodes, but not peripheral blood, marrow and spleen. Comorbidities included DNA-negative chronic inactive hepatitis B and beta-blockers-controlled hypertension. He was given, under lamivudine prophylaxis, six courses of CHOP-21 (cyclophosphamide, doxorubicin, vincristine, prednisone) plus rituximab (six doses) up to December 19, 2019. Three more rituximab infusions were given but restaging (March 11, 2020) documented persistence of duodenal MCL (Fig. 1). From March 13, the patient developed mild evening fever (single spike of 38.9 °C), responsive to azithromycin, without cough and breathing problems (Fig. 2a). On March 17, due to increasing COVID-19 rates in our region, he underwent nasopharyngeal swab and serological testing for SARS-CoV-2, which were both negative, along with a clear chest x-ray imaging. Up to March 29, the patient remained at home without respiratory symptoms and a single fever spike. He lived outside areas of COVID-19 clusters, denied any travel/contact history, and was admitted for salvage treatment on March 30, 2020. Physical examination was unremarkable and most laboratory indexes including hemogram, lactic acid dehydrogenase, serum immunoglobulins (IGs), renal function tests, liver enzymes, pro-calcitonin, creatine phosphokinase, troponin and coagulation parameters, were within limits. Differently, he had an absolute lymphocyte count (ALC) of 0.5 × 10^9^/L, including a CD4+ T cell count of 370 × 10^6^/L and elevated inflammatory indexes (C-reactive protein, 70.6 mg/L; erythrocyte sedimentation rate, 66 mm/hr). Few hours thereafter, a mild febrile peak (37.9 °C) was accompanied by onset of a moderate dyspnea and a progressive worsening of peripheral oxygen saturation (SpO2) to 67%. Arterial blood gas analysis showed PaO_2_ and pCO_2_ of 71.6 and 33 mm-Hg, respectively. Chest x-ray evidenced a bilateral interstitial infiltrate with right middle to lower lobe peripheral consolidations. Shortly after empirical antimicrobial treatment, the patient became apyretic and noninvasive ventilation (NIV) continuous positive airway pressure (CPAP; ≥5 cmH_2_O) progressively improved respiratory indexes (PaO_2_ 90.1 mm-Hg and pCO_2_ 39,2 mm-Hg). Serological testing for SARS-CoV-2 IgG/IgM was negative but nasopharyngeal swab evidenced viral RNA. Oxygenation (PaO_2_/FiO_2_ = 140), clinical and CT imaging data were consistent with COVID-19 and mild acute respiratory distress syndrome (ARDS) (Fig. 2a-c). He was started on enoxaparin (6000 IU q12 hrs), hydroxycholoroquine (200 mg b.i.d) and continued NIV support. Serological and molecular testings evidenced a progressive increase in viral load and SARS-CoV-2 IgG, while IgM remained undetectable. The clinical course progressively deteriorated to severe ARDS (PaO_2_/FiO_2_ = 50, on Apr 4), with a stepwise increase of ferritin and IL-6 serum levels (Fig. 2d-e). The patient deceased on the 16th day from hospitalization.

## Discussion

We have presented here a detailed description of the atypical COVID-19 dynamics occurred in a patient with MCL. While working case definitions for COVID-19 include acute and severe respiratory symptoms, typically with fever, unusual clinical presentations are increasingly being reported. Our patient had a protracted pre-symptomatic phase, without high-grade fever and respiratory symptoms, followed by a rapidly progressive phase and an asynchronous seroconversion pattern. Among risk factors for severe COVID-19, beyond age and male sex, our patient had mild hypertension, but never received angiotensin-converting enzyme inhibitors, and 1 week before admission his ALC was of 1.5 × 10^9^/L. Yet, his respiratory parameters quickly deteriorated with progressive lymphopenia, increasing viral load and development of the typical cytokine storm of critical patients with COVID-19.

The incubation period for COVID-19 is between 2 and 14 days and > 97% of patients develop symptoms within 11 days from infection [12]. Our patient had negative viral and serological testings 14 days before and was asymptomatic up to admission. Seroconversion times for anti-SARS-CoV-2 IgG and IgM are estimated in 11 and 12 days from symptoms onset, respectively [13]. Our patient developed IgGs 3 days after first occurrence of respiratory symptoms but IgMs were never detected up to 15 days after COVID-19 diagnosis. He was then a multiple ‘outlayer’ as to usual disease dynamics. While laboratory results might have been affected by technical issues, we emphasize that all testings were obtained through certified referral laboratories of Italian COVID-19 network (Fig. 2).

How underlying MCL and anti-lymphoma treatments might have contributed to such unusual clinical course remains subject of speculation, but a few issues are worth consideration. First, MCL may involve disease-related deficiencies in CD4+ T-cells, as in our case, resulting in impaired anti-viral immunity [10, 14]. Second, while we could have missed a late IgM rise, due to early death of our patient, the immune context of MCL might have in turn played a role. Asynchronous seroconversion (IgM later than IgG) or lack of IgM response have been described in COVID-19 [13], but disease-related deficiencies in IgM production are otherwise present in MCL [15]. In addition, use of the anti-CD20 antibody rituximab, with or without chemotherapy, is typically associated with impaired humoral responses to influenza vaccines [11, 16, 17]. Such impairment, persisting long after treatment discontinuation, is linked to a severe depletion of CD27+ memory B-cells that directly correlates with low IgM levels and impaired response to influenza vaccines [18]. Our case was SOX11-negative, suggesting origin from precursors, close to memory B cells [19].. Whether, lymphoma-associated derangements of residual memory B-cell compartments might have played a role in COVID-19 dynamics in our patient remains conjectural.

It has been reported that cancer patients, who received antineoplastic treatments within 1 month from testing, displayed a significantly lower seroconversion rate as compared to non-oncologic subjects with COVID-19 [20]. Unfortunately, no specific data on patients with NHL was presented. In contrast, a persisting SARS-CoV-2 viremia has been recently described in NHL patients exposed to rituximab, including one MCL case, while other reports have highlighted the possible association of protracted and complicated clinical course of COVID-19 in lymphoma patients who received anti-CD20 antibodies [21–25]. Such reports, although describing a limited number of patients, concordantly support that rituximab-induced B-cell depletion may concur to generate an impaired humoral response towards SARS-CoV-2 leading to ineffective viral clearance. Furthermore, it is to underline that disease-related immunodeficiencies and anticancer agents employed in combination with anti-CD20, may in turn have had a role in disturbing anti-SARS-CoV-2 immune responses in these subjects [9, 26, 27]. In this regard, exposure to Rituximab may then carry different COVID-19-related risk profiles, depending on the nature of underlying disease (i.e. autoimmune conditions vs. lymphoma) also given the significant heterogeneity in treatment schedules, intensity of anti-CD20 administration and previous treatments history [28, 29]. As an example, in patients with systemic autoimmune diseases Rituximab is usually administered as two 1000 mg flat doses given 2 weeks apart, while in thrombocytopenic purpura scheduling usually consists of four weekly infusions of rituximab at a dose of 375 mg/m^2^ [30–32]. Moreover, corticosteroids and or/methotrexate can be concurrently administered [32]. In NHL, anti-CD20 antibodies are always dosed at 375 mg/m^2^ and given, together with alkylators-containing chemotherapy, at a three-weekly frequence for six courses followed, in the case of indolent NHLs, by a maintenance phase of bi-monthly administrations for 2 years [33]. Therefore the concept of ‘anti-CD20 pre-exposure’, needs to be thoroughly assessed in the context of the above variables, to figure out the actual COVID-19-related risk profiles of a given patient.

Specific studies in patients with NHL, pre-exposed to or receiving rituximab, are then urgently needed to ascertain how anti-CD20 antibodies may interfere with the generation on an effective anti-SARS-CoV-2 humoral immune response, especially under the light of the availability of different vaccination strategies [34].

While the major limitation of this report is its single-case nature, we highlight that patients with MCL who received previous immuno-chemotherapy may develop an unusual and unfavorable clinical course upon SARS-CoV-2 infection, along with an impaired seroconversion pattern. COVID-19 screening strategies for clearing access of asymptomatic cancer patients to treatments are still heterogeneous worldwide, and, due to resources availability or local guidelines, sometimes still rely on serological testings [35]. Since patients with MCL should not be denied or delayed effective treatments, our report my prompt the adoption in these subjects of serial RT-PCR testing from nasopharyngeal samples regardless of the presence of overt symptoms and a negative viral serology.

Finally, we wish to propose that patients heavily exposed to anti-B cell therapeutic antibodies need a customized vaccination/re-challenge strategy aimed at boosting, beyond adaptive humoral responses, also innate and cellular immune systems [36]. In this regard, current development of self-replicating mRNA vaccines, may represent a way to enhance protective T cell immunity in these patients [36, 37].

## Data Availability

Data and clinical files are available from the corresponding author and investigators from participating Institutions.
